# Gene Therapy: Novel Approaches to Targeting Monogenic Epilepsies

**DOI:** 10.3389/fneur.2022.805007

**Published:** 2022-06-21

**Authors:** Kimberly Goodspeed, Rachel M. Bailey, Suyash Prasad, Chanchal Sadhu, Jessica A. Cardenas, Mary Holmay, Deborah A. Bilder, Berge A. Minassian

**Affiliations:** ^1^Division of Child Neurology, Department of Pediatrics, University of Texas Southwestern, Dallas, TX, United States; ^2^Center for Alzheimer's and Neurodegenerative Diseases, University of Texas Southwestern, Dallas, TX, United States; ^3^Department of Research and Development, Taysha Gene Therapies, Dallas, TX, United States; ^4^Division of Child and Adolescent Psychiatry, Department of Psychiatry, Huntsman Mental Health Institute, University of Utah, Salt Lake City, UT, United States

**Keywords:** genetic epilepsy, AAV9, Lafora, SLC13A5, SLC6A1, gene therapy (GT)

## Abstract

Genetic epilepsies are a spectrum of disorders characterized by spontaneous and recurrent seizures that can arise from an array of inherited or de novo genetic variants and disrupt normal brain development or neuronal connectivity and function. Genetically determined epilepsies, many of which are due to monogenic pathogenic variants, can result in early mortality and may present in isolation or be accompanied by neurodevelopmental disability. Despite the availability of more than 20 antiseizure medications, many patients with epilepsy fail to achieve seizure control with current therapies. Patients with refractory epilepsy—particularly of childhood onset—experience increased risk for severe disability and premature death. Further, available medications inadequately address the comorbid developmental disability. The advent of next-generation gene sequencing has uncovered genetic etiologies and revolutionized diagnostic practices for many epilepsies. Advances in the field of gene therapy also present the opportunity to address the underlying mechanism of monogenic epilepsies, many of which have only recently been described due to advances in precision medicine and biology. To bring precision medicine and genetic therapies closer to clinical applications, experimental animal models are needed that replicate human disease and reflect the complexities of these disorders. Additionally, identifying and characterizing clinical phenotypes, natural disease course, and meaningful outcome measures from epileptic and neurodevelopmental perspectives are necessary to evaluate therapies in clinical studies. Here, we discuss the range of genetically determined epilepsies, the existing challenges to effective clinical management, and the potential role gene therapy may play in transforming treatment options available for these conditions.

## Introduction

While 20–30% of epilepsies are acquired nongenetically, 70–80% are due to 1 or more genetic factors (1). Developmental and epileptic encephalopathies (DEE) are rare disorders characterized by early-onset, refractory seizures that occur in the context of developmental regression or plateauing. DEE are severe and difficult to treat and may result from a single gene mutation that causes gain-of-function (2) or loss-of-function epilepsy (3, 4). Monogenic epilepsies may be autosomal recessive (e.g., EPM2A/B or *SLC13A5*), autosomal dominant (e.g., *CHRNA4*), autosomal haploinsufficiency (e.g., *SLC6A1*), or X-linked (e.g., *ARHGEF9*) (5–10). Further, different pathogenic variants in the same gene may result in different epilepsy phenotypes, as seen in the *KCNQ2* gene, where the R213W variant causes benign familial neonatal seizures, and the R213Q variant causes neonatal epileptic encephalopathy with severe pharmacoresistant seizures (11).

Precision medicine describes a rational treatment strategy that is highly specific and aims to address the underlying cause of disease (12). One avenue of precision medicine involves the selection of a therapy that is directed toward modulating or bypassing the dysfunction caused by the underlying genetic defect (12). In the era of gene therapy, avenues that may be applied to epilepsy syndromes include treatments that aim to restore cellular function such as gene replacement therapy (GRT) for disorders due to loss-of-function pathogenic variants (13, 14); genetic substrate reduction therapy (gSRT) [reviewed in Coutinho et al. (15)] to reduce the overproduction of substrates; or transcriptional enhancement, designed to upregulate endogenous expression of a given gene *via* the introduction of regulatory elements (16, 17). Monogenic epilepsies are of particular interest for precision medicine, as simplified GRT, gSRT, and transcriptional enhancement therapies are promising in ameliorating disease. Here, we will focus specifically on Lafora disease, *SLC13A5* deficiency disorder (SDD), and *SLC6A1*-related disorder (SRD).

## Clinical Care

Current treatment approaches focus on treating the epilepsy syndrome *via* antiseizure medications, diet, and/or neurostimulation, rather than the underlying genetic basis of disease (9). Combinations of antiseizure medications may be necessary to achieve adequate seizure control. Further, patients may become refractory to antiseizure medications over time (18) and for some patients, specific antiseizure medications are contraindicated, as they may exacerbate neurodevelopmental disability associated with their specific epilepsy syndrome (19). Ketogenic (high fat/low carbohydrate) diets and vagus nerve stimulation approaches also have been attempted in patients with inadequate seizure control, however, with limited success (20–25). Notably, there are no currently approved treatments that address the underlying cause of disease for genetic epilepsies, presenting an urgent need for the community and an opportunity for novel approaches such as GRT and gSRT.

## Historical Context

### Advances in Genetic Diagnosis

Prior to modern genetic approaches, epilepsies were examined for their genetic basis in families using gene mapping and applied linkage analysis. The first discoveries in the 1990s identified ion channels and led to the “channelopathy” hypothesis that suggested that ion channel defects were a common underlying cause of epilepsy (1). Additionally, it is now recognized that other single-gene pathogenic variants contribute to seizure disorders.

Starting in the late 2000s, next-generation sequencing has increasingly led to discovery of pathogenic variants in specific genes and microdeletions resulting in epilepsies (26). Commercially available epilepsy panels are available to test for many genetic epilepsies.

Still, many genetic epilepsies and their natural histories are not well understood. The prognosis for genetic epilepsies is often not promising, and there is a need for innovative solutions to improve patient outcomes. In addition to the development of novel pharmaceuticals, genetic epilepsies may be approached *via* gene therapy.

### Advances in Gene Therapy

The first successful human trial of gene therapy occurred in 1990 (27). The field has rapidly expanded in the twenty-first century. One approach is GRT, which utilizes a vector such as adeno-associated virus (AAV) serotype 9 (AAV9), to deliver a functional copy of a gene to correct loss-of-function pathogenic variants, including recessive disorders (e.g., *SLC13A5*) and haploinsufficiencies (e.g., *SLC6A1*) (7, 9, 13). One example is the recent FDA approval of a gene therapy product to treat spinal muscular atrophy—a rare disease that causes infant mortality—which was the first gene therapy approval for children >2 years of age (28). There are also AAV9-based gene therapies in neurodevelopmental disorders in clinical trials (NCT02362438) following promising preclinical results (13).

The gSRT approach may utilize an AAV vector to deliver small interfering RNA that will reduce the overproduction of substrates (15). For example, the *GYS1* gene may be knocked down to prevent the overproduction of the substrate glycogen, which accumulates to cause Lafora disease (16, 29). Transcriptional enhancement approaches may be effective in haploinsufficiencies such as Dravet syndrome, where 1 allele of the *SCN1A* gene possesses loss-of-function pathogenic variants, and the other normal endogenous allele can be modified to increase its expression levels (17). These approaches have the potential to address the underlying cause of disease in inherited epilepsies that are the result of loss-of-function pathogenic variants and provide significant seizure relief to patients.

AAV vectors have been extensively studied for treatment of central nervous system (CNS) diseases (30). AAV9, specifically, is a vector with great potential for treating neurological disorders, as it crosses the blood-brain barrier and targets CNS neurons (31). While other viral vectors transduce neurons, AAV9 is the most studied AAV vector for CNS disorders, and there is more clinical evidence of safety, efficacy, and stability of gene transfer to the CNS with this serotype than with other vectors (32).

Further, to aid in the development of next-generation gene therapy technologies for diagnosis of genetic epilepsies, a better understanding of natural history of disease will be required and is addressed in the next section. These studies inform clinical development and help identify outcome measures for clinical investigation.

## Clinical Trial Readiness

Studies into the natural history of disease are essential to understanding how diseases progress and to inform drug development so that researchers and clinicians can have strong metrics available to evaluate how best to demonstrate efficacy and, ultimately, improve patients' quality of life. Regulatory agencies are increasingly acknowledging the importance of natural history data in the context of rare disease and gene therapy drug development, having released draft guidance on the topics in recent years (33). Natural history studies, although informative, may not accurately represent disease populations due to factors such as study design, variability of supportive care practices, changes in medical care or terminology over time, selection bias, etc. In monogenic epilepsies, due to the relatively recent identification of genetic causes, may particularly be lacking in a detailed and longitudinal understanding of the disease course. Animal models, therefore, also have an important role to play in understanding disease progression. Animal models currently exist for some, but not all, of the recessive and haploinsufficient epilepsies (see Table 1 for examples of available models) but may not fully replicate the clinical phenotype, which represents a challenge to characterizing the outcomes of potentially disease-modifying investigational drugs. While electroencephalography findings in animal models are comparable to those in humans, neurologic and motor deficits do not always correspond well with the human disease. GRT and gSRT approaches utilizing AAV vector technology may address diseases resulting from pathogenic variants in single genes (13, 15). In particular, AAV9 has shown promise for treating neurological disorders as it crosses into the brain and infects neurons (31). In the following sections, this review will highlight 3 monogenic inherited diseases, areas of active research by our groups: Lafora disease, SDD, and SRD, as well as their clinical picture, mouse models, and approaches to gene therapy for each condition.

**Table 1:** Potential monogenic epilepsy candidates for gene therapy.

**Disorder**	**Gene**	**Protein**	**Protein function**	**Most common seizure type**	**Mouse model**
Dravet syndrome	*SCN1A^#^*	Na_v_1.1	Voltage-gated sodium channel (34, 35)	GTCS (36)	*Scn1a +/–* (35)
EIEE (8)	*SLC13A5*	NaCT	Plasma membrane sodium-dependent citrate transporter (37–39)	Clonic or Tonic (40)	*Slc13a5* KO (41)
	ARHGEF9*	Collybistin	GABA receptor clustering at inhibitory synapses (42)	GTCS (5)	*Arhgef9* KO (5)
	*WWOX*	WWOX	Development and function of CNS (43)	GTCS (44)	*Wwox* KO (43)
Familial infantile myoclonic epilepsy or EIEE	*TBC1D24*	TBC1D24	Vesicle trafficking for neuronal signal transmission (45)	Myoclonic or clonic seizures (46)	S324Tfs*3 (45)
Lafora—PME	*EPM2A*	Laforin	Glycogen phosphatase (47)	GTCS (48)	*Epm2a* KO (47)
	*EPM2B*	Malin	Ubiquitin E3 ligase (47)		*Epm2b* KO (47)
Pyridoxine dependent epilepsy	*ALDH7A1*	ALDH7A1	Lysine catabolism (49)	Focal Seizures (50)	*Aldh7a1* KO (49)
*SLC6A1*-related disorder	*SLC6A1^#^*	GAT-1	Sodium- and chloride-dependent GABA transporter (51)	Absence seizures (52)	*Slc6a1* KO (51)

* *X-linked.*

# *Haploinsufficiency.*

*All others are autosomal recessive.*

*CNS, central nervous system; EIEE, early infantile epileptic encephalopathy; GABA, gamma-aminobutyric acid; GAT, GABA transporter; GTCS, generalized tonic-clonic seizures; KO, knockout; PME, progressive myoclonus epilepsy*.

### Lafora Disease

Lafora disease is a severe, fatal, autosomal recessive progressive myoclonus epilepsy (PME) that results from accumulation of Lafora bodies, abnormal glycogen aggregates (6). Two genes are now known to be involved in Lafora disease: *EPM2A* and *EPM2B* (48, 53–56). Loss-of-function pathogenic variants in *EPM2A* or *EPM2B* lead to an accumulation of Lafora bodies (an abnormal form of glycogen that cannot be metabolized) and subsequent Lafora disease (47).

#### Presentation and Progression

The mean age of Lafora disease onset is 13.4 years (57). Patients with classical Lafora disease develop normally until adolescence, when they present with action and stimulus-sensitive myoclonus, in addition to tonic-clonic and absence seizures (48). At presentation, it is challenging to distinguish Lafora disease from idiopathic generalized epilepsies (48). Thus genetic testing is critical, as it reveals pathogenic variants in the *EPM2A* and *EPM2B* genes (58).

Patients most often receive antiseizure medications, namely valproic acid, which is typically effective at suppressing seizure activity, however the treatment is palliative (59). Lafora patients quickly develop symptoms of dementia and intractable seizures (57). Patients tend to lose autonomy by 6 years after disease onset and die from status epilepticus, aspiration pneumonitis, or other complications of neurodegenerative disease within 10 years of disease onset (57).

To date, only one large-scale natural history study for Lafora disease exists, suggesting more studies are needed to describe the heterogenous disease and inform clinical investigation more fully (57).

#### Gene Therapy Development

It has been shown that *Epm2a* knockout (KO) and *Epm2b* KO mouse models replicate essential features of Lafora disease, such as neuronal degeneration and accumulation of Lafora bodies in muscle, liver, and brain (47, 60, 61). Recently, a proof-of-concept paper demonstrated that a viral vector carrying clustered regularly interspaced short palindromic repeats (CRISPR)/Cas9 with a guide RNA could be used to target and cut the *Gys1* gene responsible for producing brain glycogen that leads to Lafora bodies and Lafora disease. In this study, neonatal *Epm2a* KO and *Epm2b* KO mice were injected intracerebroventricularly with an AAV9 vector targeting *Gys1* that led to an editing rate of 17% of *Gys1* alleles. The effect of this editing was a 50% reduction in GYS1 protein, decreased glycogen accumulation, and decreased neuroinflammatory markers (47). This approach addresses the underlying cause of disease using a gene editing strategy, but alternative approaches such as a simpler gene delivery system without CRISPR/Cas9 may have a better safety profile and greater clinical potential.

### *SLC13A5* Deficiency Disorder

Pathogenic variants in the gene *SLC13A5* impair the sodium/citrate cotransporter, NaCT, with subsequent elevation in plasma and CSF citrate levels (62). These variants result in an autosomal recessive epileptic encephalopathy known as SLC13A5 deficiency disorder as SDD. *SLC13A5* pathogenic variants were first identified in 2014 when whole-exome sequencing was performed in 3 individuals with similar clinical presentation of epileptic encephalopathy from 2 families (7). Whole-exome sequencing is one approach now used to detect SDD (63). Additionally, *SLC13A5* is included in some commercially available epilepsy panels.

#### Presentation and Progression

Beginning within the first week of life most patients present with seizures and later often have status epilepticus (7, 64). However, there is phenotypic variability, and some patients have onset of seizures later in infancy. Patients with SDD may progress to lifelong drug-resistant epilepsy, with most seizures being convulsive (65). Seizure severity may decrease with age and some patients may even reach seizure freedom (40, 65). Broad-spectrum antiseizure medications often reduce seizure frequency, but targeted treatments are lacking and further innovation is needed.

Affected individuals show global developmental delay with intellectual disability and poor speech and communication (23). Patients often develop significant motor impairments and deficits in cognitive and expressive language (65). Patients typically have persistent neurological symptoms including ataxia, abnormal muscle tone, and abnormal involuntary movements (65). Additionally, patients with SDD may later develop dental enamel hypoplasia (65). It is possible for patients to live well into adulthood (65).

To date, there have been no published natural history studies for SDD. However, one natural history study of SDD is underway (NCT04681781), suggesting more studies may be needed to describe the disease state and inform clinical investigation more fully.

#### Gene Therapy Development

An *Slc13a5* KO model has been utilized to investigate SLC13A5 disease pathology. It has demonstrated myoclonic and nonconvulsive focal seizures as seen in patients, but with no obvious behavior or pathological abnormalities (66). Recently, a self-complementary AAV9 vector carrying a *SLC13A5* gene was developed (37). Preliminary data showed that delivery of this gene therapy to cerebrospinal fluid in young adult *Slc13a5* KO mice resulted in rescue of epileptic activity. Additionally, treated KO mice had lower plasma citrate levels compared with KO mice that did not receive GRT (37). This approach addresses the underlying cause of the disease, and the clinical potential is under investigation.

### *SLC6A1*-Related Disorder

*SLC6A1* pathogenic variants were first identified in 2015 when 2 truncations and 4 missense pathogenic variants were found in patients with epileptic encephalopathies with myoclonic-atonic seizures (67). *SLC6A1* is included in some commercially available epilepsy panels. *SLC6A1* pathogenic variants cause a haploinsufficiency of sodium- and chloride-dependent gamma-aminobutyric acid transporter type-1 (GAT-1), resulting in SRD (65).

#### Presentation and Progression

The mean age of seizure onset is ~2.5 years of age in patients with SRD (9). Sixty percent of patients had developmental delay before seizure onset (9). The most prevalent epilepsy syndromes associated with SRD are myoclonic-atonic seizures (24%), genetic generalized epilepsy (23%), and non-acquired focal epilepsy (10%) (9). Further, it was found that absence seizures were the most common type of seizures in SRD (9). Common clinical features are epilepsy, developmental delay or cognitive impairment, and autistic traits (9). In addition patients may develop hypotonia, language disorder, and sleep issues.

Most patients require a care team consisting of neurologists, developmental pediatricians, genetic counselors, and speech and occupational therapists (9). Due to limited clinical data for SRD, treatment is determined based on the presenting clinical epilepsy syndrome and typically includes broad-spectrum antiseizure medications (9).

To date, there have been few natural history studies for SRD (52, 67, 68), indicating more studies are needed to describe the disease state and inform clinical investigation more fully.

#### Gene Therapy Development

*Slc6a1* KO mice have been used to model seizure activity (51). These mice partially recapitulate human SRD as they have tremors, abnormal gait, reduced strength, absence seizures, anxious behavior, and cognitive impairment (9). *SLC6A1* is a potential candidate for gene therapy because it results from pathogenic variants that cause haploinsufficiency, thereby allowing for gene replacement or transcriptional enhancement strategies to potentially alleviate the burden of disease. However, no gene therapy studies have been published on SRD.

### Opportunity for Gene Therapy in Monogenic Epilepsies

GRT for CNS disorders has led to promising preliminary safety and efficacy data in clinical trials (31). gSRT has shown promising results preclinically, but additional work is needed in the clinic (16). Single-injection approaches of viral vectors may lead to a safe and effective strategy in the clinic (31). Importantly, these strategies address the underlying cause of disease and have the potential to stabilize the progression of the disease. However, there is still a need for preclinical proof-of-concept research for gene therapy applications for monogenic epilepsies in animal models. Important endpoints to track patient progress and measure success for gene therapy for genetic epilepsies are survival, seizure susceptibility, the number of recurrent seizures, biomarkers such as citrate levels in SDD, and adverse events (37). The development and application of appropriate outcome measures is vital to lead to the next generation of medicines for persons with monogenic epilepsies.

In contrast to targeting the gene underlying the monogenetic epilepsy, an alternative approach may be used, such as gene therapy delivering an AAV vector for an engineered voltage-gated potassium channel to drive down neuronal excitability and thereby reduce seizure (69). Another approach is to virally overexpress neuropeptide Y, which has been shown to suppress seizures in animal models (70). These approaches are not precision medicine addressing the underlying cause of disease, and their clinical applicability must be tested.

## Remaining Challenges in The Clinical Development Path Forward For Gene Therapies

### Seizure

By addressing the underlying cause of disease, gene therapy has the potential to impact disease course more than treating seizures alone. Seizure reduction will remain an important clinical goal for patients with epilepsy, yet clinicians rely upon patient and caregiver reports of seizure activity, which are known to have limited reliability (71). Furthermore, nocturnal seizure frequency is inherently difficult to capture through self- or parent-reporting. Reporting and monitoring of seizure activity is therefore often inadequate. Seizures themselves may not be the best target for genetic epilepsies, as they can vary in frequency and severity depending in part on the patient subpopulation. In some genetic epilepsies such as SDD, there may be a reduction in seizures, but continued morbidity due to developmental disabilities, including impairments in motor and cognitive abilities (65). Cognitive dysfunction may result from the underlying disease process itself, which gene therapies may address (72).

### Developmental Concerns

In monogenic epilepsies, patients with DEE may miss or have delayed developmental milestones (7) that can negatively impact quality of life and capacity for achieving independent living. These motor and cognitive delays may affect functioning (7) and merit a means of systematic measurement and ongoing monitoring to inform the evaluation of treatment response. Early initiation of gene therapy for genetic epilepsies may mitigate or prevent the development of motor and cognitive manifestations of the diseases. For example, there is a growing body of evidence that patients with a degenerative motor neuron disease, spinal muscular atrophy, treated pre-symptomatically with GRT achieve improved motor outcomes compared to patients treated later in the disease course presumably by preventing or slowing neuronal loss (73).

Motor dysfunction such as hypotonia, stereotypies, and ataxia impair mobility and purposeful use of movement (7, 9). Motor impairment and global developmental delay may be apparent in infancy, such as in EIEE, or may manifest with severe, progressive deterioration following normal development, as experienced by children with Lafora disease (62, 74). It is therefore important to expand our understanding of the spectrum of motor impairments affecting patients with monogenic epilepsy and establish endpoints related to motor ability. Such endpoints would indicate clinical meaningful changes and be applicable across multiple monogenic epilepsy syndromes with early childhood onset.

Cognitive dysfunction, which can result from both recurrent seizure activity and the underlying disease process itself (72), has substantial impact on patient quality of life. It requires that clinicians consider metrics for improving not only seizure frequency and severity but also cognitive function. To this end, more research is needed to understand progressive cognitive decline in epilepsy, especially as the disease course in some genetic epilepsies shows a reduction in seizures, but a continued progression of cognitive decline.

Autism spectrum disorder may also accompany intellectual disability in patients with genetic epilepsies such as Dravet syndrome, and has a substantial impact on a patient's potential to achieve independence (75). There is a need for clearer neurodevelopmental/neurophysiological endpoints to track a patient's developmental abilities both accurately and efficiently over time. It will be important to identify endpoints that can characterize developmental trajectories associated with specific conditions. Such endpoints could subsequently provide an early indication of treatment response when patients' trajectories shift following intervention.

## Discussion

Advances in genetic technologies have identified a growing number of monogenic genetic epilepsies potentially amenable to gene therapies. The state of AAV-based gene therapy has advanced a great deal with extensive study of AAV9 in preclinical models and in the clinic. Loss-of-function pathogenic variants may be highly amenable to gene therapy, namely by GRT and gSRT, which address the underlying cause of disease without the need for gene editing. However, there is still need for translational research to advance new therapeutics to the clinic. Understanding of disease progression through natural history studies may be an important precursor to interventional studies as meaningful clinical endpoints are highly dependent upon the severity and rapidity of clinical decline. Preclinical animal models may also be important to inform optimal timing of dosing relative to disease progression, as rapidly lethal diseases like Lafora disease may have a narrow therapeutic window. While SDD and SRD have different underlying pathology and less severe epilepsy outcomes than Lafora disease, early intervention may be critical in intervention strategies to improve cognitive, behavioral, and functional measures and the chance for good quality of life and greater independence from caregivers. Study design, clinical endpoints, dose selection, inclusion/exclusion criteria, and safety all need to be carefully considered in order to best serve patients.

## Author Contributions

DB, SP, MH, and BM supported conceptualization of the paper, and reviewed and revised the manuscript. CS, JC, RB, and KG reviewed and revised the manuscript. All authors approved the final draft for submission.

## Funding

This work was funded by the National Institutes of Health under award P01NS097197 and Taysha Gene Therapies. BM holds the University of Texas Southwestern Jimmy Elizabeth Westcott Chair in Pediatric Neurology and is Chief Medical Advisor at Taysha Gene Therapies. Medical writing and editorial support were provided by Kelly A. Hamilton, PhD, of AlphaScientia, LLC, and funded by Taysha Gene Therapies.

## Conflict of Interest

DB is a consultant for Encoded Therapeutics, BioMarin Pharmaceuticals, and Synlogic Therapeutics. KG has provided consultation to Jaguar Gene Therapies. RB is an inventor on patents that have been licensed to various biopharmaceutical companies and for which she may receive payments. The authors declare that this study received funding from Taysha Gene Therapies. The funder had the following involvement in the study: MH, SP, CS, and JC are employees of Taysha Gene Therapies; KG and BM receive salary and research support from Taysha Gene Therapies; RB has sponsored research agreements with Taysha Gene Therapies; and DB is a member of the scientific advisory board for Taysha Gene Therapies. Each author was involved in the review, revision, and approval of the manuscript. UT Southwestern holds equity in Taysha Gene Therapies, which is a licensee of UTSW technology.

## Publisher's Note

All claims expressed in this article are solely those of the authors and do not necessarily represent those of their affiliated organizations, or those of the publisher, the editors and the reviewers. Any product that may be evaluated in this article, or claim that may be made by its manufacturer, is not guaranteed or endorsed by the publisher.
